# *Haemonchus contortus* P-Glycoproteins Interact with Host Eosinophil Granules: A Novel Insight into the Role of ABC Transporters in Host-Parasite Interaction

**DOI:** 10.1371/journal.pone.0087802

**Published:** 2014-02-03

**Authors:** Mohamed Issouf, Fabrice Guégnard, Christine Koch, Yves Le Vern, Alexandra Blanchard-Letort, Hua Che, Robin N. Beech, Dominique Kerboeuf, Cedric Neveu

**Affiliations:** 1 INRA, UMR1282 Infectiologie et Santé Publique, Nouzilly, France; 2 Université François Rabelais de Tours, UMR1282 Infectiologie et Santé Publique, Tours, France; 3 Institute of Parasitology, McGill University, Quebec, Canada; UCSD, United States of America

## Abstract

Eosinophils are one of the major mammalian effector cells encountered by helminths during infection. In the present study, we investigated the effects of eosinophil granule exposure on the sheep parasitic nematode *Haemonchus contortus* as a model. *H. contortus* eggs exposed to eosinophil granule products showed increased rhodamine 123 efflux and this effect was not due to loss of egg integrity. Rh123 is known to be a specific P-glycoprotein (Pgp) substrate and led to the hypothesis that in addition to their critical role in xenobiotic resistance, helminth ABC transporters such as Pgp may also be involved in the detoxification of host cytotoxic products. We showed by quantitative RT-PCR that, among nine different *H. contortus* Pgp genes, *Hco-pgp-3, Hco-pgp-9.2, Hco-pgp-11* and, *Hco-pgp-16* were specifically up-regulated in parasitic life stages suggesting a potential involvement of these Pgps in the detoxification of eosinophil granule products. Using exsheathed L3 larvae that mimic the first life stage in contact with the host, we demonstrated that eosinophil granules induced a dose dependent overexpression of *Hco-pgp-3* and the closely related *Hco-pgp-16*. Taken together, our results provide the first evidence that a subset of helminth Pgps interact with, and could be involved in the detoxification of, host products. This opens the way for further studies aiming to explore the role of helminth Pgps in the host-parasite interaction, including evasion of the host immune response.

## Introduction

Soil transmitted helminths have a major impact on both human and animal health. These parasites are responsible for increased morbidity, mortality and premature birth [1]–[4]. While the host immune response against parasitic helminths is highly complex, granulocytes are known to be involved in both the initiation and effector immune response phases [5], [6]. These responses are associated with a proliferation of T-helper 2 lymphocytes, plasma cells, eosinophil cells, basophils and mast cells [7], [8]. Among these granulocytes, eosinophils constitute a homogeneous population of cells that are recruited from bone marrow into the blood and then host tissues where they survive for several days or even weeks [6]. *In vitro* studies have demonstrated that granulocytes interact with pathogenic helminths causing major damage to the parasites [9]. During degranulation, eosinophils release cationic proteins with significant cytotoxic activity such as the major basic protein, eosinophil peroxidase and eosinophil-derived neurotoxin [10]. In this respect, it is expected that successful establishment of the parasite *in vivo* might require efficient detoxification mechanisms to defend against the host immune response products. To date, these mechanisms remain largely unknown in helminths.

Pgps are membrane pumps involved in the active transport of many biological and xenobiotic substances using the energy provided by ATP hydrolysis. They are of particular interest here since vertebrate Pgps have been shown to be involved in the transport of immune cell products [11]–[16]. For example, inhibition of Pgp activity in natural killer cells has been correlated with a decrease in their cytotoxicity [17]. We have a precedent from research into resistance to the anthelmintic drug ivermectin, to expect that Pgps from the parasite may play a similar role to those in the host and so provide protection by the transport of host granulocyte products [18]–[20].

The sheep parasitic nematode *Haemonchus contortus* represents one of the most pathogenic species impacting the livestock industry [21]. Exposure of infective *H. contortus* larvae to host eosinophils *in vitro* reduces their ability to establish a subsequent infection *in vivo*, making this species an appropriate model to investigate the interaction beetween eosinophils and the parasite [22]. We have previously developed an egg based assay where fluorescent rhodamine-123 transport out of *H. contortus* eggs is mediated by Pgp [23], [24]. In this study, we observed that eosinophil granule proteins increase this Rh123 transport, suggesting a role for Pgp in protection of the parasite. In an effort to identify the molecular mechanisms involved, we cloned sequences for several different Pgp genes from *H. contortus*. Quantitative PCR was used to investigate the expression kinetics of Pgp genes in different developmental stages in order to identify those that could potentially be involved in host product detoxification *in vivo*. We also examined changes in Pgp gene expression induced by exposure to eosinophil granules *in vitro*.

Here, we report for the first time, that several *H. contortus* Pgps show increased expression associated specifically with the parasitic stages and that two of these show a specific increase in expression following exposure to host granulocyte products.

## Results

### Isolation of sheep eosinophils and granule product purification

**Figure 1:**
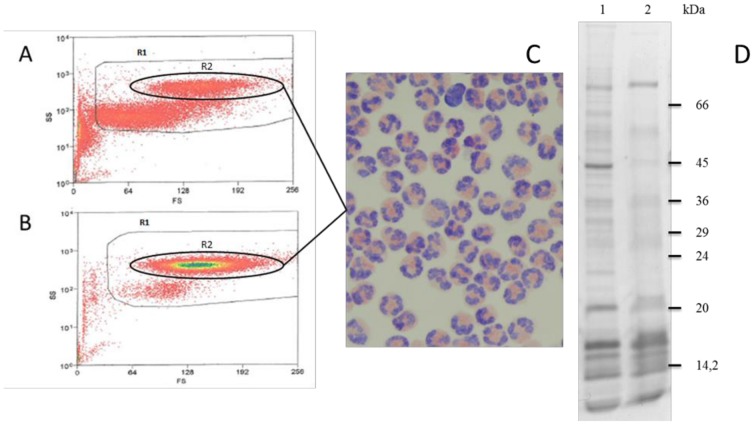
Purification of eosinophil cells from sheep infected with *H. contortus*. Granulocyte cell populations were isolated from the leukocyte population by flow cytometry using Forward Scatter (FS) and Size Scatter (SS) parameters. The dot-plot histograms with total leukocyte population (A) and dot-plot histogram after Percoll density separation (B) highlight the R2 enriched cell population correponding to eosinophils. Purity of eosinophil cell population was further checked by Giemsa May-Grunwald (GMG) staining (C). The GMG stains the eosinophil cell nuclei in purple and cytoplasmic granules in orange. Total eosinophil lysate (1) and purified eosinophil granule proteins (2) were separated by 10% polyacrylamide gel electrophoresis and stained with Coomassie blue (D).

We combined the Percoll gradient method with flow cytometry assays in order to select an intact eosinophil population from the blood of infected sheep (figure 1A). This approach allowed us to obtain a cell population containing approximately 95% eosinophils (figure 1B). Purity of the eosinophils was further checked by Giemsa May-Grunewald staining (figure 1C). These purified cells enabled us to extract eosinophil granule proteins by ultracentrifugation for subsequent experiments, including functional analysis *in vitro* and modulation of Pgp mRNA expression. Purified granule proteins were analyzed by SDS-PAGE highlighting approximately 10 distinct bands, ranging from 11 to 70 kDa. In contrast, total eosinophil lysate used as a control for granule purification efficiency contained several additional proteins ranging from 11 to over 100 kDa (figure 1D).

### Eosinophil granule products modulate *H. contortus* Pgps activity

**Figure 2:**
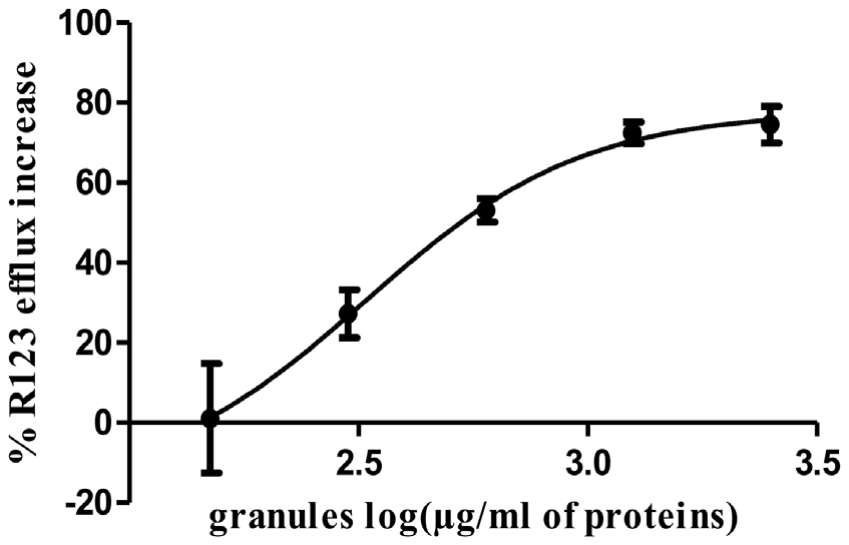
Rhodamine Rh123 efflux assay performed on *Haemonchus contortus* eggs stimulated with eosinophil granule proteins. The regression line was obtained from the log agonist (granule proteins) versus response variable-slope model using Prism software. A significant effect of eosinophil granule products on Pgp activation was observed (P<0.0069).

We have previously reported that Pgp activity can be analyzed in *H. contortus* eggs using a rhodamine (Rh123) efflux assay [23], [24]. In the present work, Rh123 efflux increased proportionally with exposure to granule proteins (figure 2). This relationship was established up to a protein concentration of 1250 µg/ml, beyond which a plateau of 75% stimulation was reached (P<0.0069). The loss of Rh123 from the eggs was not due to a loss of egg integrity since FITC was excluded from both untreated eggs and those exposed to eosinophil granule products, whereas FITC was easily able to enter eggs damaged by freezing (figure S1). This result provided the first functional evidence that parasite Pgp activity can be modulated by host eosinophil granules products.

### Identification of Pgp transcripts in the adult stage of *H. contortus*

**Figure 3:**
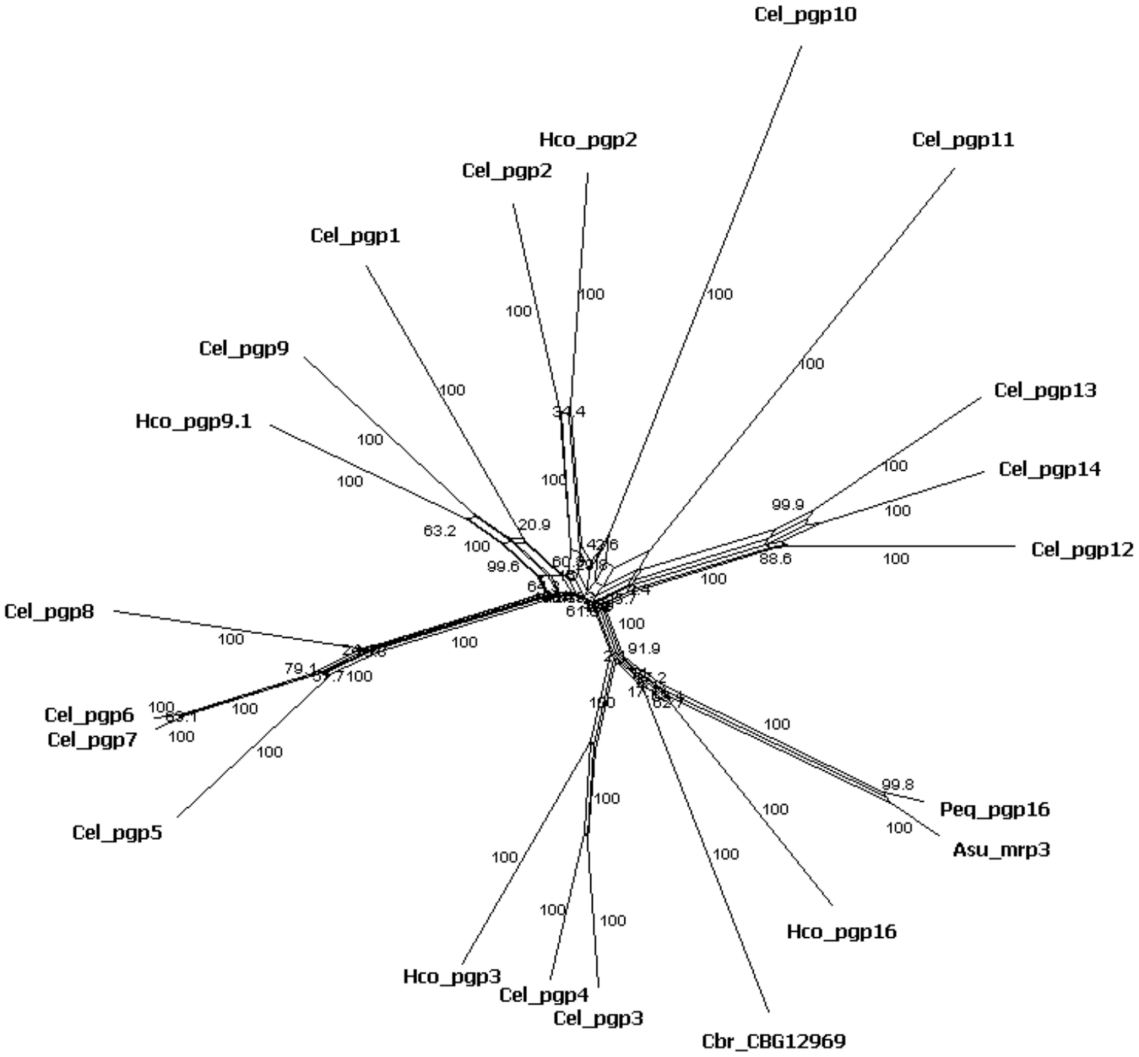
Split-decomposition phylogenetic network including *C. elegans* Pgp sequences, *Hco-pgp-3*, *Hco-Pgp-16* and their closest homologs. Numbers over the branches represent bootstrap values for 1000 pseudoreplicates. The three letter prefixes in Pgp gene names Hco, Cel, Cbr, Peq and Asu refer to *H. contortus, C. elegans, C. brigsae, Parascaris equorum* and *Ascaris suum* respectively. Genbank-accession numbers for nucleotide sequences are provided in Table S3.

In the present work 3' RACE-PCR experiments allowed the identification of 8 partial cDNA sequences corresponding to the 3′ end from distinct *H. contortus* Pgp transcripts. All these cDNA sequences were then further extended in their respective 5′ end by RT-PCR and among the eight candidates, four complete coding cDNA sequences have been obtained. Sequence descriptions including size and closest homologs in *C. elegans* and other nematode species are summarized in table 1. When the resulting complete, or partial, sequences matched unambiguously with a single orthologous gene in *C. elegans*, the corresponding *H. contortus* Pgp cDNA sequences were named following Beech *et al* [25]. No clear ortholog of *Hco-pgp-16* could be identified in *C. elegans* and so the numerical series of Pgp genes was extended. Interestingly, we have identified three distinct *H. contortus* Pgp sequences for which the closest homolog in *C. elegans* was *Cel-pgp-9* (i.e. *Hco-pgp-9.1*, *Hco-pgp-9.2* and *Hco-pgp-9.3*). Phylogenetic analyses of the complete sequences obtained in *H. contortus* were performed including homologous sequences in the closest species (*Caenorhabditis elegans*, (Cel), *Caenorhabditis briggsae* (Cbr), *Ascaris suum* (Asu), *Parascaris equorum* (Peq)). A split-decomposition network analysis revealed the phylogenetic proximity of the *Hco-pgp-3* sequence with the *Cel-pgp-3*/*Cel-pgp-4* group (figure 3). *Hco-pgp-9.1* and*Cel-pgp-9* are clearly orthologous genes. *Hco-pgp-16* clustered with *Peq-pgp-16*, *Asu-mrp-3* and *Cbr-CBG12369*, suggesting the *Ascaris* sequence has been misidentified. Even though the *Hco-pgp-16* sequence has no ortholog in *C. elegans*, it clustered within the *Cel-pgp-3* and *Cel-pgp-4* group. An alignment of the Hco-PGP-16 deduced amino-acid sequence with Cel-PGP-4 and Cel-PGP-3 revealed some amino acid similarity ranging from 66 to 67% respectively (figure S2)

**Table 1:** Summary of the cDNA sequences corresponding to P-glycoproteins identified in adult *H. contortus*.

Name	Accession number	Size of amplified cDNA (bp)	Top blastx match	% of similarity	Top blastx match to *C. elegans* Pgp genes
*Hco-pgp-2*	AF003908 [27]	4175	CON-PGP-2 AGJ71178.1	94%	CEL-PGP-2 NM_059306.4
*Hco-pgp-3*	JX430936	4115	CON-PGP-3 AGJ71177.1	87%	CEL-PGP-3 NM_077500.3
*Hco-pgp-9.1*	JX430937	4169	CBN-PGP-9 EGT49138.1	81%	CEL-PGP-9 NM_075086.2
*Hco-pgp-9.2*	JX430938	1703	CBN-PGP-9 EGT49138.1	85%	CEL-PGP-9 NM_075086.2
*Hco-pgp-9.3*	JX430935	911	CBN-PGP-9 EGT49138.1	85%	CEL-PGP-9 NM_075086.2
*Hco-pgp-10*	JX430939	1741	CEL-PGP-10 NM_076804.3	88%	CEL-PGP-10 NM_076804.3
*Hco-pgp-11*	JX430940	866	OVO-PGP AAD49436.1	60%	CEL-PGP-11 NM_063273.3
*Hco-pgp-14*	KF192604	600	CBN-PGP-14 EGT46666.1	96%	CEL-PGP-14 NM_077727.6
*Hco-pgp-16*	JX430941	3884	PEQ-PGP-16 AGL08023.1	74%	NO CLEAR HOMOLOG

### A subset of Pgp genes is specifically up-regulated during the parasitic phase of *H. contortus* life cycle

In order to provide information on *H. contortus* Pgps that could potentially be involved in the host-parasite interaction, we investigated their expression kinetics through different developmental stages of the parasite. *H. contortus* presents a simple life cycle, including a free living and a parasitic phase within a single host. Eggs released in host faeces develop through three successive larval stages (L1 to L3) on the pasture. The transition to parasitism occurs when the host ingests infective third stage larvae (L3) while grazing. Then, larvae exsheath (xL3) and develop through a fourth larval stage (L4) into mature adult worms into the host abomasum.

The expression level of 9 different *Hco-pgp* mRNA (including *Hco-pgp-2*) was investigated in eggs, L3, L4 and adult male stages using real-time PCR. Note that adult females were not included as they contain eggs that could interfere with Pgp expression analysis.

**Figure 4:**
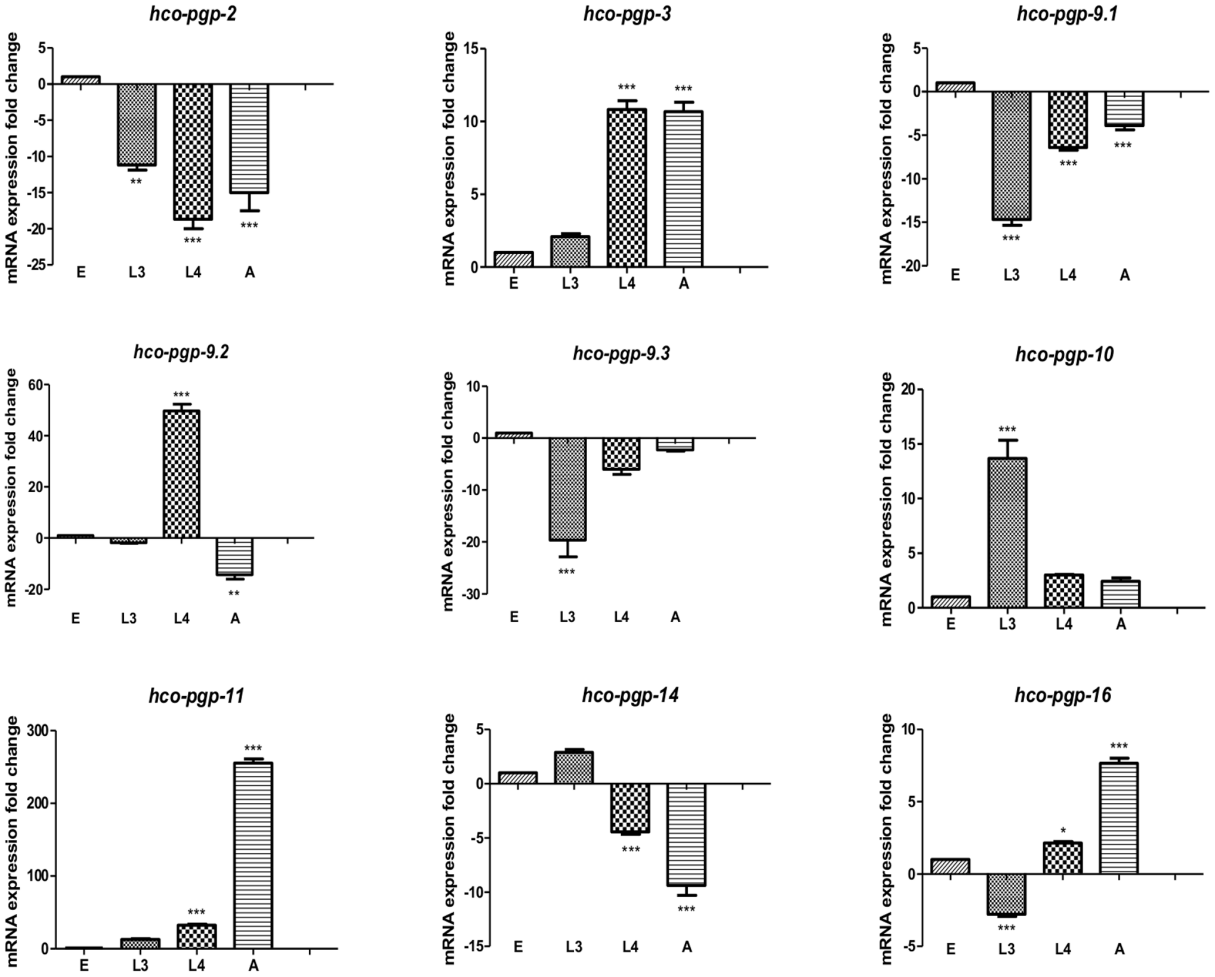
Expression of Pgp mRNAs during *H. contortus* life cycle. Real-time RT-PCR experiments were performed in triplicate for each developmental stage: Eggs (E) and third stage larvae (L3) corresponding to the free-living stages as well as in fourth stage larvae (L4) and adult males (A) corresponding to parasitic stages. The mRNA expression levels observed in the eggs was normalized to 1. The mRNA fold changes were calculated using three distinct reference genes (*gapdh, actin* and *β-tubulin*). For each sample, real-time RT-PCR were performed in triplicate and repeated twice using two independent cDNAs templates. * Significant difference from control values after Bonferroni's Multiple Comparison Test (* P<0.05; ** P<0.01; *** P<0.001).

All the Pgp genes were found to be expressed in the four different developmental stages studied in this work. However, for some Pgps striking differences of expression level have been observed between the free-living and the parasitic stages (figure 4). For example, *Hco-pgp-3*, *Hco-pgp-9.2*, *Hco-pgp-11* and *Hco-pgp-16* transcripts were found to be significantly more abundant in parasitic life stages (L4 and/or adult male) in comparison with free-living stages (eggs and/or L3) with an increased expression ranging from 7.5-fold (*Hco-pgp-16*) up to 250-folds (*Hco-pgp-11*). Within the parasitic stages, *Hco-pgp-3* was found to be equally expressed in L4 and adult whereas *Hco-pgp-11* and *Hco-pgp-16* mRNA were found to be more abundant in adult stage than in L4. Among the three *H. contortus Pgp-9* homologues it is noteworthy that only *Hco-pgp-9.2* was found to be temporarily up regulated in the L4 stage in contrast with *Hco-pgp-9.1* and *Hco-pgp-9.3* that were found to be more abundantly expressed in free living stages. *Hco-pgp-3*, *Hco-pgp-9.2*, *Hco-pgp-11* and *Hco-pgp-16* represent candidates of major interest for their potential involvement in host product detoxification *in vivo* even though we cannot rule out that other Pgps may also play an important role in the host parasite interaction. These four Pgps were selected for further characterization to investigate their potential interaction with eosinophil granule products.

### Eosinophil granules induce the specific overexpression of *Hco-pgp-3* and *Hco-pgp-16* genes in exsheathed L3 larvae

**Figure 5:**
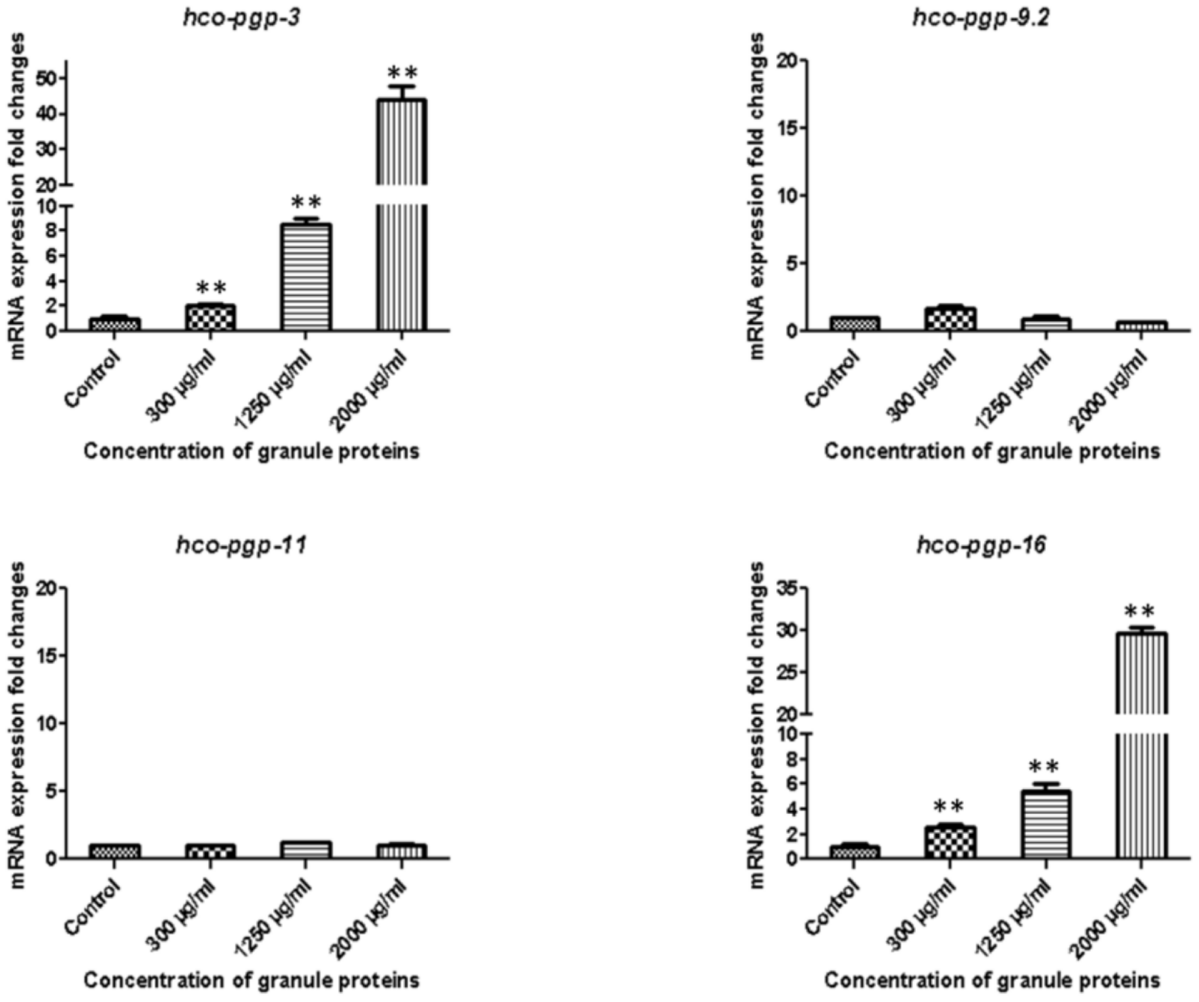
Expression of Pgp mRNAs in xL3 larvae of *H. contortus* after 24 hours stimulation with increasing concentrations of eosinophil granule proteins. Real-time RT-PCR experiments were performed in triplicate for each sample using two distinct cDNA preparations per sample. mRNA expression levels were normalized using non-stimulated xL3. The mRNA fold changes were calculated using three distinct reference genes (*gapdh, actin* and *β-tubulin*). ** Significant difference from control values after Man-Whitney test analyses (*P<0.01*).

The Rh123 transport assay data suggest that eosinophil granule products may interact directly with Pgp transporters in *H. contortus* eggs. Previous studies of bacteria, mammals and nematodes report that Pgp substrates can specifically induce overexpression of their corresponding Pgp gene [26]–[29]. Taking advantage of this property, we examined which *H. contortus* Pgp genes respond specifically to host granule products *in vivo*. Expression of *Hco-pgp-3*, *Hco-pgp-9.2*, *Hco-pgp-11* and *Hco-pgp-16* was monitored by qRT-PCR in artificially exsheathed L3 *H. contortus* larvae that mimic the first parasitic stage in contact with eosinophil granules *in vivo*. Larvae were exposed for 24 h to increasing concentrations of granule products ranging from 300 to 2 000 µg/ml. Comparisons between the stimulated and non-stimulated exsheathed L3 (control) showed a significant (*P<0.01, n = 6*) and dose dependent increase of mRNA expression levels of *Hco-pgp-3* and *Hco-pgp-16* in stimulated larvae, whereas expression of *Hco-pgp-9.2* and *Hco-pgp-11* remained unchanged (figure 5). This result shows that eosinophil granule products contain compounds that specifically modulate the expression of *Hco-pgp-3* and *Hco-pgp-16* and suggests these as candidate Pgps that specifically protect *H. contortus* from granule products *in vivo*.

## Discussion

Helminth Pgps have been widely studied for their involvement in xenobiotic transport and their association with drug resistance. Their implication in other critical functions such as the host-parasite interaction remained to be explored. We found that eosinophil granule products specifically affected the transport of Rh123 that has previously been shown to be mediated by Pgp transporters. Eosinophils are known to be key effectors during helminth infection and we hypothesized that parasitic helminth Pgp could play a role in detoxification of eosinophil granule products. Eosinophil granule products are mainly composed of four highly basic proteins that have been shown to be toxic for helminths. These include eosinophil peroxidase (EPO), major basic protein (MBP), eosinophil cationic protein (ECP), and eosinophil-derived neurotoxin (EDN) [30], [31]. Eosinophil granule proteins are stored in a crystalloid structure that can be purified by ultracentrifugation [32]. However purification of an eosinophil cell population from sheep blood remains challenging. For example Terefe *et al.* [22] reported that a single Percoll gradient (1.090 g/ml) allowed the purification of sheep eosinophils with a purity ranging from 43 to 63%. In the present work, we optimized this approach using two gradient Percoll densities in combination with cell sorting that resulted in a cell population containing 95% eosinophils. Thus, we could hypothesize that most of the protein collected after eosinophil lysis and ultracentrifugation would correspond to granules products. Separation of purified sheep eosinophil granule protein with SDS-PAGE revealed a set of proteins ranging from 11 to 20 kDa. It is tempting to hypothesize that at least some of them could correspond to the major cationic proteins (MBP, ECP and EDN) presenting similar molecular weight as reported for human eosinophil granule proteins (i.e. 13, 16 and 18 kDa respectively) [33]. These granule proteins were then used in a Rh123 efflux assay to assess their potential role as modulators of Pgp activity. Using *H. contortus* eggs, we showed that eosinophil granule proteins are able to specifically interact with the parasite Pgp. This result provides functional evidence that granule proteins could represent potential substrates for the parasite Pgp *in vivo*. Interestingly, the relationship between granule protein concentration and the resulting Rh123 efflux is similar to those observed with a classical Pgp substrates such as colchicine [34] suggesting a strong interaction between *H. contortus* Pgps and granule proteins. Therefore this result provided a robust argument to further investigate this interaction at the molecular level.

In order to identify *H. contortus* Pgp genes potentially involved in granule protein transport *in vivo* our first objective was to explore their expression in a parasitic stage (adult male) of *H. contortus*. In contrast with mammals, nematodes possess a large diversity of Pgp genes. The model nematode *Caenorhabditis elegans* that is closely related to *H. contortus* encodes up to 14 Pgp genes (*pgp-1* to *pgp-14*) [35]. However, in *H. contortus*, only a single complete Pgp sequence (*Hco-pgp-2*) has been reported to date [36]. In a recent study, Williamson *et al* developed quantitative PCR for several of these, corresponding to partial transcript sequences identified in the infective larval stage (L3), providing a basis for *H. contortus* Pgp expression analysis [37]. In the present work, *H. contortus Pgp* transcripts have been further investigated in the adult stage using an RT-PCR approach. We have also extended transcript sequence information including 3'UTR regions in order optimize transcript specific primer design for subsequent quantitative PCR experiments.

In the present study we found corresponding partial or complete cDNA sequence for 8 of them (i.e. *Hco-pgp-3*, *Hco-pgp-9.1*, *Hco-pgp-9.2*, *Hco-pgp-9.3*, *Hco-pgp-10*, *Hco-pgp-11*, *Hco-pgp-14* and *Hco-pgp-16*) and further confirmed the expression of the previously reported *Hco-pgp-2* [36] in *H. contortus* adult males.

Analyses of the *H. contortus* Pgp sequences found a high degree of conservation with their respective homologs in *C. elegans*, with the striking exception of *Hco-pgp-16*, for which the closest homolog found in Genbank corresponds to the recently reported *pgp-16* sequence from the distantly related horse parasite *Parascaris equorum*. Despite this lack of a clear ortholog within the free living species *C. elegans*, the closest homolog in *H. contortus* was the *Hco-pgp-3* sequence. It is noteworthy that the complete cDNA sequence corresponding to *Hco-pgp-3* gene identified in the present work perfectly overlaps with two distinct partial cDNA sequences previously designated as *Hco-pgp-3* (HM635768) and *Hco-pgp-4* (HM635766) stressing the need to obtain complete cDNAsequence before assigning a definitive nomenclature of *H. contortus* genes. Interestingly, we were able to identify three potential homologs of *C. elegans Pgp-9* that mostly differ in their respective 3′UTR region (data not shown). The origin and functional implication of such potential duplication events remain to be explored.

For parasitic nematodes, the transition to parasitism implies some major adaptations such as evasion of the host immune response and changes in nutrition, metabolism and growth [38], [39]. In order to identify *H. contortus* Pgp genes potentially involved in host-parasite interaction and more specifically those involved in eosinophil granule product detoxification, we have compared the expression level of *H. contortus* Pgps in free living stages (eggs and L3) and parasitical stages (L4 and adult). Our results showed that *Hco-pgp-9.2*, *Hco-pgp-11*, *Hco-pgp-3* and *Hco-pgp-16* mRNAs are each overexpressed in L4 and/or adult in comparison with to free living stages.

Homologs of *pgp-9* and *pgp-11* have been associated with anthelminthic resistance in the parasitic nematodes *Teladorsagia circumcincta* and *Parascaris equorum*, respectively [40], [41]. Over-expression of these genes in parasitic stages of *H. contortus* raises the question of potential functions during the interaction between host and parasite. The closest homolog of *Hco-pgp-3* identified in databank is *C. elegans pgp-3*, which is involved in defense against natural toxins produced by plants and bacteria [42], [43]. Interestingly, our phylogenetic analysis revealed that *pgp-16* orthologs of *H. contortus*, *Ascaris suum* and *Parascaris equorum* are clustered within the same group as *Cel-pgp-3*, highlighting the importance of this Pgp group for future studies.

It is tempting to speculate that the specific over expression of *Hco-pgp-3*, *Hco-pgp-9.2*, *Hco-pgp-11* and *Hco-pgp-16* in parasitic stages might reflect their potential activation during the host/parasite interaction. It has been reported that Pgp substrates can induce over expression of their cognate Pgp genes in nematodes [28], [29] and we were able to show that, *Hco-pgp-3* and *Hco-pgp-16*, but not *Hco-pgp-9.2* or *Hco-pgp-11*, were specifically induced in exsheathed L3 (xL3) larvae exposed to eosinophil granule protein in a dose dependent manner. The specific induction of *Hco-pgp-3* and the closely related *Hco-pgp-16* represent an attractive and promising result suggesting that a subset of *H. contortus* Pgps might be involved in detoxification of host immune cell products. This lays the basis for future work aiming to decipher in more detail the mode of action of fractionated eosinophil proteins and other host products on parasitic helminth Pgps.

In conclusion, the study of nematode Pgps has, until now, been mainly based on their involvement in the detoxification of drugs in the rather artificial setting of anthelmintic treatment. The present work demonstrates for the first time that interactions occur between parasitic helminth Pgps and host cell products and provides some new insight about potential molecular mechanisms involved in overcoming the host immune response. This in turn may provide novel potential targets for the future control of *H. contortus* and other helminths of medical and veterinary importance.

## Materials and Methods

### Ethics statement

All animal experiments were approved by the Regional Centre-Limousin Ethics Committee (CL2006-012) and conducted under a license issued by the Directorate of veterinary services in Tours; France with the accreditation number: B-37-175-3.

### Purification of eosinophils

Eosinophils from the peripheral blood of *Haemonchus contortus* infected sheep (21 days post-infection) were purified by discontinuous density Percoll gradients in combination with flow cytometry sorting. Briefly, 600 ml of blood were collected and mixed immediately with 50 ml of a solution containing ethylene diamine tetra-acetic acid (EDTA) at a concentration of 40 mg per ml in order to avoid blood coagulation. In order to lyse the erythrocyte cells, 150 ml of blood were mixed with 275 ml of water with stirring. After 20 s, cell lysis was stopped by adding 275 ml of 2,5X PBS solution. The cells were then centrifuged at 400 g for 5 min and the pellet washed 3 times in 50 ml of 1X PBS (0.14 M NaC1, 8 mM Na2HP04, 3 mM KCI, l.5 mM KH2P04. pH 7.4). A multiple discontinuous density gradient (1.040, 1.055, 1.070 and 1.080 g/ml) was prepared. The Percoll solutions were poured in a 15 ml centrifuge tube starting with the solution of the lowest density (1.040 g/ml) and finishing with the solution of the highest density (1.080 g/ml). The cell suspension was carefully layered on top of the gradient and centrifuged at 400 g for 15 min. The purified cells were collected at the 1.070/1.080 g/ml interface and washed with a 1X PBS. Isolated cells were further purified by flow cytometry (MoFlo®, DakoCytomation, 4850 Innovation Drive, Fort Collins, CO) using Forward Scatter (FS) and Size Scatter (SS) parameters. The SS is a 90 degree light scatter that reflects the granularity of the cells and the forward angle light scatter (FS) is proportional to the cell size. The purity of the cell population was further checked by Giemsa May-Grunwald (Reactifs RAL^®^, Martillac, France) (GMG) staining according to the manufacture's recommendation.

### Isolation of granules

The granules were isolated by ultracentrifugation according to the methods described by Slifman *et al.* [44]. In brief, the eosinophil cells were centrifuged at 600 g for 10 min and the resulting pellet was washed in 5 ml of 1X PBS. Eosinophils were then lysed in 0.25 M sucrose by repeated passage through an 18 gauge needle attached to a syringe. Eosinophil lysate was centrifuged at 600 g for 10 min in order to separate unbroken cells and granules. Granules were then pelleted by ultracentrifugation at 10 000 g for 10 min. The quantity of granule proteins was determined using a nanodrop spectrophotometer (Thermo Scientific, Waltham, Massachusetts, USA) and store at −80°C until used.

### Denaturing Sodium Dodecyl-sulfate Polyacrylamide Gel Electrophoresis (SDS-PAGE)

Eosinophil lysate and purified eosinophil granules proteins were separated on SDS-PAGE acrylamide gels and stained with Coomassie blue [45]. Both samples were suspended in denaturation buffer and heated at 100°C for 5 min and loaded onto 10% polyacrylamide gels. Proteins were visualized after gel staining in a 0.25% Coomassie blue solution.

### Parasites

For all experiments, the “Weybridge” *Haemonchus contortus* isolate was used. Sheep were infected with 6 000 infective third stage larvae (L3). Eggs were collected 28 days post infection from host faeces as previously described [46]. The L3 larvae were harvested from coprocultures and subsequently artificially exsheathed (xL3) following exposure to 0.1% hypochlorite solution for 10 min. Fourth stage larvae (L4) and adult male nematodes (A) were collected from the abomasal mucosa of sheep necropsied after 7 or 25 days post-infection respectively.

### Rhodamine 123 (Rh123) assay

The effect of eosinophil products on parasite Pgp activity was determined by changes in the accumulation of rhodamine (Rh123) in the *H. contortus* eggs. Approximately fifteen thousand eggs were suspended in deionized water (1 ml) and were centrifuged at 1 000 g for 1 min. The supernatant was discarded. Rh123 solution (2 ml of 1.5 µM Rh123 in deionized water) was added to the egg pellet and incubated for 5 min at 20°C in the dark. Eosinophil granule products were then added at four distinct final concentrations (300, 600, 1250 and 2 500 µg/ml of granule proteins) and the eggs were incubated for a further 10 min at 20°C before being centrifuged at 1 000 g for 1 min. The supernatant was discarded and the eggs were washed three times with 5 ml of ice cold deionized water. Eggs were kept in the dark for 60 min before analyses. Specific fluorescence was measured using a Quanta Master spectrofluorometer (PTI, NJ, USA) equipped with a 75 W xenon lamp (λ excitation  = 495 nm, λ emission  = 525 nm). Data were given in arbitrary units of green fluorescence corrected for the native green fluorescence of eggs and expressed as the percentage of fluorescence in control eggs. Three replicates per condition were analyzed. The Rh123 efflux without eosinophil granules was normalized to 0. The Hill equation was used to describe the dose-response relationships [47] by calculating the following equation:

Y = Bottom + (Top – Bottom)/(1+10∧ ((logEC50-X)))

where Bottom is the baseline and Top is the maximum effect, EC50 the dose giving half the maximum effect, X the granule protein concentration (log). Bottom values were adjusted to 0, corresponding to the fluorescence in the control eggs.

### Molecular Biology

In order to identify *H. contortus* Pgps, total RNA was extracted from 10 adult males. Frozen parasite pellets were homogenized in Trizol reagent (Invitrogen, Carlsbad, California, USA) and RNA was isolated according to the manufacturer's recommendations. RNA pellets were dissolved in 35 µl of RNA secure re-suspension solution (Ambion, Austin, Texas, USA). DNA was removed form RNA samples using RQ1 RNase-Free DNase (Promega, ). The RNA concentrations were measured using a nanodrop spectrophotometer (Thermo Scientific, Waltham, Massachusetts, USA) and adjusted to 100 ng/µl.

First strand cDNA synthesis was performed on 1 µg of total RNA using the oligo (dT) RACER primer (Invitrogen) and the superscript III reverse transcriptase (Invitrogen,Carlsbad, California, USA) according to the manufacturer's instructions. Using first strand cDNA prepared from adult *H. contortus*, the 3′ ends of Pgp cDNAs were isolated by 3′RACE PCR using the GeneRacer kit (Invitrogen, Carlsbad, California, USA) according to the manufacturer's recommendations. 3'RACE PCR amplicons were obtained with forward primers designed upon *H. contortus* supercontig sequences (available at http://www.sanger.ac.uk/cgi-bin/blast/submitblast/h_contortus) In order to extend sequence in their 5' ends, a new set of PCR experiments was performed using either the SL1 primer (GGTTTAATTACCCAAGTTTGAG) or forward primers designed from supercontig sequences. The complete list of primers is provided in the table S1. All PCR reactions were performed with a programmable thermocycler (Biometra, Gottingen, Germany). 3′ RACE experiments were carried out in a final volume of 25 µl containing 15 ng of first-strand cDNA, 0.5 units of LongAmp™ *Taq* polymerase (New England Biolabs, Ipswich, United Kingdom), 200 µM of each dNTP and 0.4 µM of each primer. The reaction mixture was denatured by heating to 94°C for 30 sec, followed by 32 cycles at 94°C for 30 sec, 54–60°C (depending on specific melting temperature of primers) for 30 sec, 72°C for 30–240 sec (depending on the amplicon size). A final extension step was performed at 72°C for 7 min. Amplification products were cloned in PGEM-T vector (Promega, charbonnieres, France) and sequenced by GATC biotech (Konstanz, Germany).

### Sequence comparison and Phylogenetic analyses

Database searches were performed using the *Haemonchus contortus* blast server (http://www.sanger.ac.uk/cgi-bin/blast/submitblast/h_contortus) and the BLAST Network Service (National Center for Biotechnology Information), using the BLASTX algorithm [48].

The nucleotide sequences (Genbank-accession numbers are provided in Table S3) were translated and amino acids sequences were aligned using MUSCLE [49]. The corresponding nucleotide alignments were obtained by concatenating codons using the REVTRANS server (http://www.cbs.dtu.dk/services/RevTrans/) [50]. The model of nucleotide substitution that best fitted the data was determined using jModeltest version 2.0 [51] on the Phylemon 2 server [52].

Phylogenetic relationships between Pgp sequences were defined for all the full length nucleotide sequences using the split-decomposition method implemented in SPLITSTREE version 4.11.3 [53] using the general time reversible (GTR) scheme of substitution with a gamma distribution model of site rates variation (jModeltest results).

### Real-time PCR

Expression of *H. contortus* Pgps in different developmental stages was analyzed using total RNA extracted from 25 µl of egg pellet, 5 000 L3, 400 L4, 10 adult males respectively using Trizol reagent (Invitrogen). In order to investigate the eosinophil granule effects on Pgp expression modulation, total RNA was extracted from xL3 incubated in water (control) or with increasing concentrations of eosinophil granule products (ranging from 300 to 2 000 µg/ml of proteins) during 24 hours. One microgram of total RNA from different nematode stages was used to generate cDNA with the oligo (dT) RACER primer (Invitrogen) and the superscript III reverse transcriptase (Invitrogen) according to the manufacturer's instructions. Quantitative RT-PCR experiments were carried out with specific forward and reverse primers designed either in identified sequences. Primer sequences used for quantitative RT- PCR experiments are provided in table S2. PCR amplification products were run on an electrophoresis gel and sequenced to ensure the absence of non-specific amplification products and that the primers were suitable for qRT-PCR.

Real-time PCR experiments were performed by monitoring the increase of fluorescence of IQ SYBR® Green in real-time (Bio-Rad, Hercules, CA, USA) with the Rotor-Gene 3000 (Corbett Research, Sydney Australia). The relative gene expression fold changes were calculated by the Gene Expression Analysis software (Bio-Rad, Hercules, CA, USA) using *H. contortus gapdh, actin*, and *β-tubulin* as reference genes. Pgp mRNA expression of eggs or xL3 incubated in the absence of eosinophil granules was normalized to 1. The data are presented as fold changes in mean ± SEM of mRNA expression. For the analysis of *H. contortus* P-glycoprotein mRNA expression in different nematode developmental stages, statistically significant differences between eggs and other stages were analyzed with one-way ANOVA test followed by Bonferroni's Multiple Comparison Test. Differences were considered significant when P<0.05. Statistically significant differences between xL3 in the presence and absence of eosinophil granules were analyzed with the non-parametric Man-Whitney test using Prism software (v5.02, GraphPad Software, San Diego, Ca, USA).

## Supporting Information

Figure S1 **Fluorescein isothiocyanate (FITC) uptake assay performed on** ***H. contortus*** **eggs following exposure to host granule products.** In order to control *H. contortus* egg viability after exposure to host eosinophil granule products, an FITC uptake assay was performed on eggs incubated in PBS (A), eggs incubated with eosinophil granule products (B), eggs frozen at −80°C. The absence of FITC uptake in the eggs incubated with or without host eosinophil granule products (A and B) confirmed their viability. In contrast, strong fluorescence associated with FITC uptake was observed in eggs previously frozen at −80°C during one hour (C). These stained eggs represent a control for egg mortality. Bars: 50 µm. Method: Approximately one thousand *H. contortus* eggs were suspended in PBS 1X and were incubated 1 h at room temperature with host granule proteins at a final concentration of 2 500 µg/ml. As a control of viability or mortality, eggs were also incubated in PBS 1X at room temperature or frozen at −80°C during one hour respectively. Fluorescein isothiocyanate (FITC) solution (20 µl of 1 mg/ml FITC in PBS 1X) was added to the eggs and incubated for 1 h at 24°C in the dark. The eggs were then washed three times with 1 ml of ice cold deionized water and analyzed by fluorescence microscopy (DP50 camera, Olympus) using a filter band-pass with 450 to 480 nm of excitation and 515 nm of emission.
(TIF)

Figure S2 **Alignment of Hco-PGP-16, Hco-PGP-3, Cel-PGP-3 and Cel-PGP-4 sequences.** Hco-PGP-16, Hco-PGP-3, Cel-PGP-3 and Cel-PGP-4 sequences were aligned using the MUSCLE algorithm [49] and further processed using the GeneDoc program. Typical features of Pgp including Walker A and Walker B motifs, ABC transporter signatures and the 12 transmembrane domains are highlighted. Amino acids common to the four sequences are shaded in dark blue. Amino acids conserved between Hco-PGP-3, Cel-PGP-3, Cel-PGP-4 but not Hco-PGP-16 are shaded in light blue. Amino acids conserved between Hco-PGP-16, Cel-PGP-3, Cel-PGP-4 but not Hco-PGP-3 are shaded in red.
(TIF)

Table S1 **Primers used for the identification of Pgp cDNAs by RT-PCR.**
(DOCX)

Table S2 **Primers used for real-time quantitative PCR assays.**
(DOCX)

Table S3 **NCBI accession numbers for nucleotide sequences used for Split-decomposition phylogenetic network.**
(DOCX)
