# Supplementary figures and images for: Haemonchus contortus P-Glycoproteins Interact with Host Eosinophil Granules: A Novel Insight into the Role of ABC Transporters in Host-Parasite Interaction
Source: PLoS One. 2014 Feb 3;9(2):e87802. doi: 10.1371/journal.pone.0087802 (PMC3912070; doi:10.1371/journal.pone.0087802)

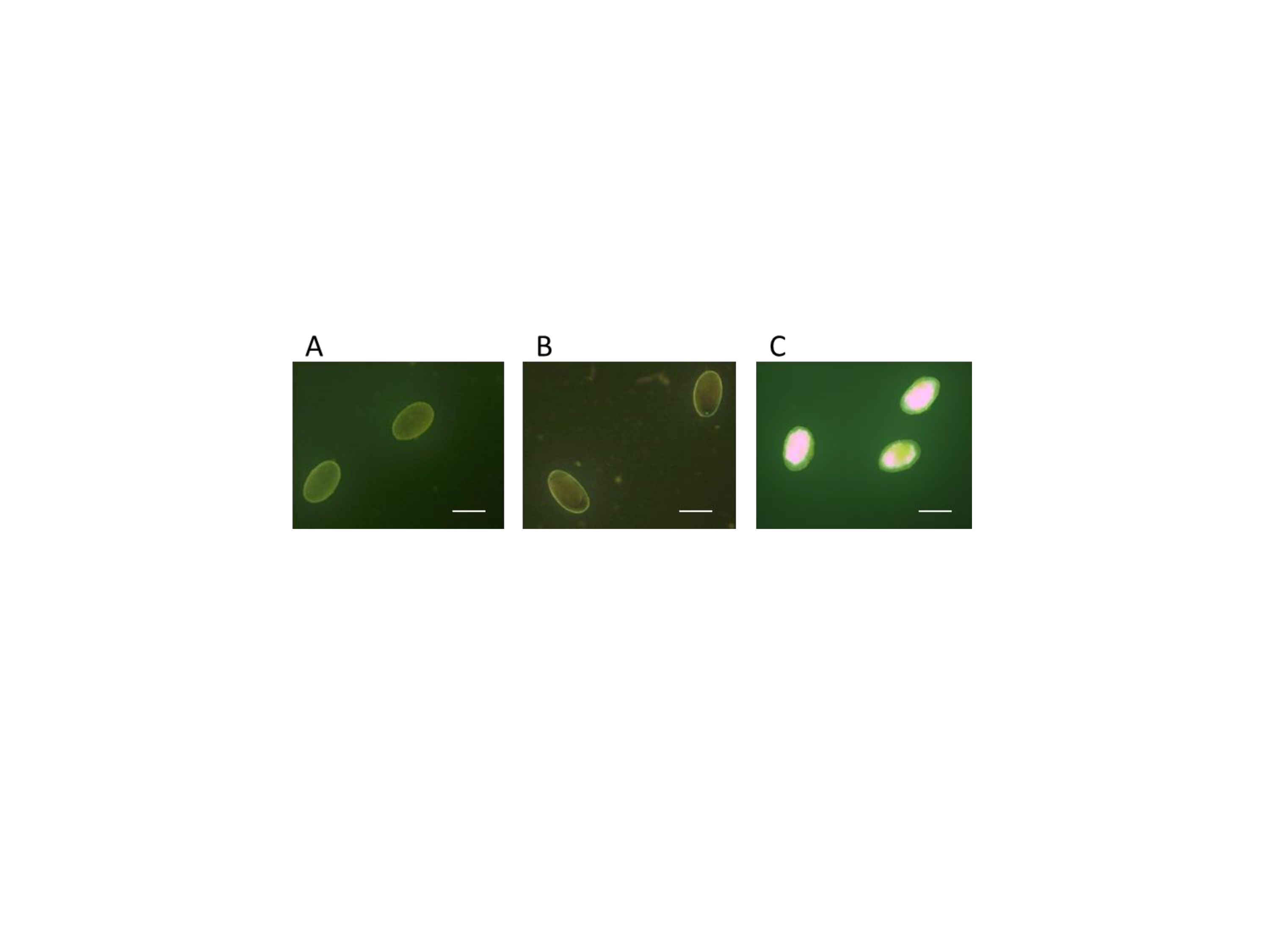

Supplement: Figure S1 — Fluorescein isothiocyanate (FITC) uptake assay performed on H. contortus eggs following exposure to host granule products. In order to control H. contortus egg viability after exposure to host eosinophil granule products, an FITC uptake assay was performed on eggs incubated in PBS (A), eggs incubated with eosinophil granule products (B), eggs frozen at −80°C. The absence of FITC uptake in the eggs incubated with or without host eosinophil granule products (A and B) confirmed their viability. In contrast, strong fluorescence associated with FITC uptake was observed in eggs previously frozen at −80°C during one hour (C). These stained eggs represent a control for egg mortality. Bars: 50 µm. Method: Approximately one thousand H. contortus eggs were suspended in PBS 1X and were incubated 1 h at room temperature with host granule proteins at a final concentration of 2 500 µg/ml. As a control of viability or mortality, eggs were also incubated in PBS 1X at room temperature or frozen at −80°C during one hour respectively. Fluorescein isothiocyanate (FITC) solution (20 µl of 1 mg/ml FITC in PBS 1X) was added to the eggs and incubated for 1 h at 24°C in the dark. The eggs were then washed three times with 1 ml of ice cold deionized water and analyzed by fluorescence microscopy (DP50 camera, Olympus) using a filter band-pass with 450 to 480 nm of excitation and 515 nm of emission. (TIF) [file pone.0087802.s001.tif]

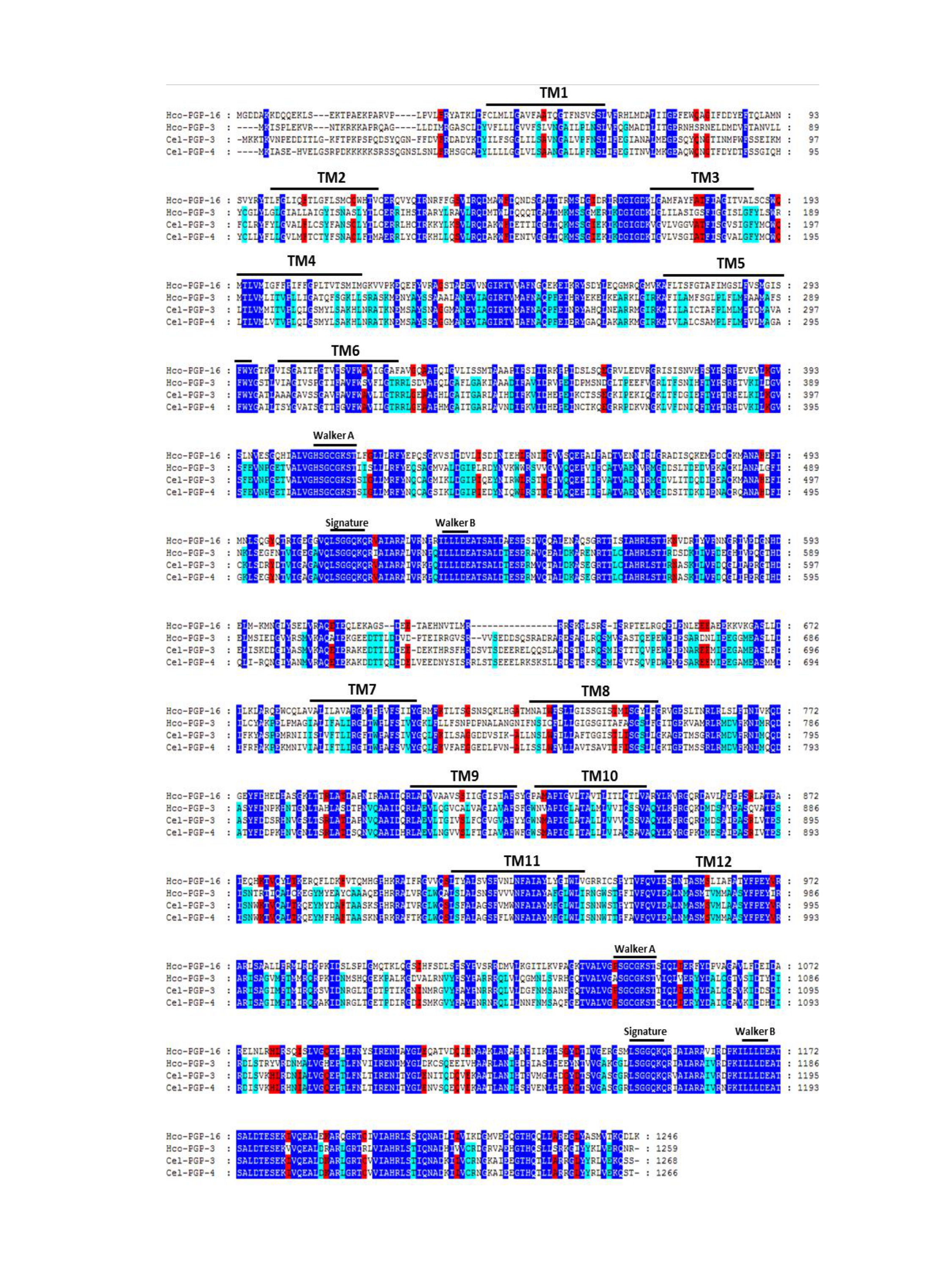

Supplement: Figure S2 — Alignment of Hco-PGP-16, Hco-PGP-3, Cel-PGP-3 and Cel-PGP-4 sequences. Hco-PGP-16, Hco-PGP-3, Cel-PGP-3 and Cel-PGP-4 sequences were aligned using the MUSCLE algorithm [49] and further processed using the GeneDoc program. Typical features of Pgp including Walker A and Walker B motifs, ABC transporter signatures and the 12 transmembrane domains are highlighted. Amino acids common to the four sequences are shaded in dark blue. Amino acids conserved between Hco-PGP-3, Cel-PGP-3, Cel-PGP-4 but not Hco-PGP-16 are shaded in light blue. Amino acids conserved between Hco-PGP-16, Cel-PGP-3, Cel-PGP-4 but not Hco-PGP-3 are shaded in red. (TIF) [file pone.0087802.s002.tif]
