# Supplementary material for: Haemonchus contortus P-Glycoproteins Interact with Host Eosinophil Granules: A Novel Insight into the Role of ABC Transporters in Host-Parasite Interaction
Source: PLoS One. 2014 Feb 3;9(2):e87802. doi: 10.1371/journal.pone.0087802 (PMC3912070; doi:10.1371/journal.pone.0087802)
Supplement: Table S1 — Primers used for the identification of Pgp cDNAs by RT-PCR. (DOCX) [file pone.0087802.s003.docx]

Table S1: Primers used for the identification of Pgp cDNAs by RT-PCR.

| Gene | Primer sequences |
| --- | --- |
| *Hco-pgp-3* | PCR3F GGTCTAACCCCAGAGGAGTTT  PCR3R GCTACTCGTCCATCACGACA  PCR3R1 TCCCTCGTCGAATACAATGAT |
| *Hco-pgp-9.1* | PCR9.1F ATGGGCTTTTTGAAGAAGAA  PCR9.1F1 ATGGTTACAACACTCGCGCA  PCR9.1R CGTCTTTCTGCCAGACTTCC |
| *Hco-pgp-9.2* | PCR9.2F CTACCGTTTTGGTTCGGTCTT  PCR9.2F1 TCGCAGGAGCCGATCCTCTTT  PCR9.2R GAACCGCTCACGTCTCTCTG |
| *Hco-pgp-9.3* | PCR9.3F GAAGTCGATGGTAACGACTTGC  PCR9.3R GTGAAAACAAGGCGGACAGT  PCR9.3R1 CAACCCAGTTCGAAAGGAAA |
| *Hco-pgp-10* | PCR10F GATCGCGATAGCTCCAGCCGAA  PCR10F1 TTGGAAAAGGGATGATGCTC  PCR10F1 GCACTCTTGTGCTCGTTCCA  PCR10R TCGTGGGTACCCTCTTCAAC |
| *Hco-pgp-11* | PCR11F ACGATCACCACGAAGCTGAACG  PCR11R TGTCCGTTGCAAGCTGCGACT  PCR11R1 CCCAAATGCACACCAGAGTG |
| *Hco-pgp-14* | PCR14F AACTCTCGGGTGGACAGAAGCA  PCR14F1 CGCTAGTTCGAGATCCCAAG  PCR14R CAGTACACGTGAAACGCAAGA  PCR14R1 ACGGCTCCGGAATTGATAG |
| *Hco-pgp-16* | PCR16F ATGGGTGACGACGCGAAA  PCR16F1 GGTGAGCGAGGCAGTATGTT  PCR16R GCAAACAAAACGATTCATCTACTT |
