# Supplementary material for: Haemonchus contortus P-Glycoproteins Interact with Host Eosinophil Granules: A Novel Insight into the Role of ABC Transporters in Host-Parasite Interaction
Source: PLoS One. 2014 Feb 3;9(2):e87802. doi: 10.1371/journal.pone.0087802 (PMC3912070; doi:10.1371/journal.pone.0087802)
Supplement: Table S2 — Primers used for real-time quantitative PCR assays. (DOCX) [file pone.0087802.s004.docx]

Table S2: Primers used for real-time quantitative PCR assays.

| Gene | Primer sequences |
| --- | --- |
| *Hco****-****actin* | ActinF ACAGGATGCAGAAAGAAATCAC  ActinR TGGACAGAGAGGCAAGGATAG |
| *Hco****-****gapdh* | GapdhF GTGTGAACCACGAGACCTACA  GapdhR TATCGTCCATGCTAGCTGGTT |
| *Hco****-****β-tubulin* | BtubF CCAATTGACGCATTCACTTG  BtubR GATCAGCATTCAGCTGTCCA |
| *Hco-pgp-2* | qPCR2F TGAAAAGATCGTGCAAGACG  qPCR2R GTCGTTGTGTTTCGCATAGC |
| *Hco-pg-3* | qPCR3F CGCTGGACACAGAAAGTGAA  qPCR3R TTCCGAGACAGAAGGGACTG’ |
| *Hco-pgp-9.1* | qPCR9.1F ATAGCTGAGGGGTGGGATG  qPCR9.1R TCTGCCAGACTTCCACCACA |
| *Hco-pgp-9.2* | qPCR9.2F CCAGTCCACCTCAATTCCAC  qPCR9.2R AACCGCTCACGTCTCTCTG |
| *Hco-pgp-9.3* | qPCR9.3F CTCTTCTTCCAATCCCACCA  qPCR9.3R CAACCCAGTTCGAAAGGAAA |
| *Hco-pgp-10* | qPCR10F ACGTCGGCCTGTTGTACTTC  qPCR10R TCGTGGGTACCCTCTTCAAC |
| *Hco-pgp-11* | qPCR11F TTTCACGAATCCGCTATCTGT  qPCR11R GAACAGTTCAGCAACGGACA |
| *Hco-pgp-14* | qPCR14F CCGTTCGGACCGTTACACTA  qPCR14R CAGTACACGTGAAACGCAAGA |
| *Hco-pgp-16* | qPCR16F AAAAGCGAGACAAGGTCGAA  qPCR16R TGTTTGGTTACCATGCTTGC |
