# Supplementary material for: Haemonchus contortus P-Glycoproteins Interact with Host Eosinophil Granules: A Novel Insight into the Role of ABC Transporters in Host-Parasite Interaction
Source: PLoS One. 2014 Feb 3;9(2):e87802. doi: 10.1371/journal.pone.0087802 (PMC3912070; doi:10.1371/journal.pone.0087802)
Supplement: Table S3 — NCBI accession numbers for nucleotide sequences used for Split-decomposition phylogenetic network. (DOCX) [file pone.0087802.s005.docx]

**Table S3 NCBI accession numbers for nucleotide sequences used for Split-decomposition phylogenetic network.**

| **Name** | **Organism** | **GenBank accession no.** |
| --- | --- | --- |
| *Asu-mrp-3* | *Ascaris suum* | JI164802.1 |
| *Cbr-pgp-CBG12969* | *Caenorhabditis briggsae* | XM_002630484.1 |
| *Cel-pgp-1* | *Caenorhabditis elegans* | NM_070012.4 |
| *Cel-pgp-2* | *Caenorhabditis elegans* | NM_059306.4 |
| *Cel-pgp-3* | *Caenorhabditis elegans* | NM_077500.3 |
| *Cel-pgp-4* | *Caenorhabditis elegans* | NM_001270214.1 |
| *Cel-pgp-5* | *Caenorhabditis elegans* | NM_001270187.1 |
| *Cel-pgp-6* | *Caenorhabditis elegans* | NM_001047822.3 |
| *Cel-pgp-7* | *Caenorhabditis elegans* | NM_077411.2 |
| *Cel-pgp-8* | *Caenorhabditis elegans* | NM_077410.4 |
| *Cel-pgp-9* | *Caenorhabditis elegans* | NM_075086.2 |
| *Cel-pgp-10* | *Caenorhabditis elegans* | NM_076804.3 |
| *Cel-pgp-11* | *Caenorhabditis elegans* | NM_077410.4 |
| *Cel-pgp-12* | *Caenorhabditis elegans* | NM_077725.3 |
| *Cel-pgp-13* | *Caenorhabditis elegans* | NM_077726.2 |
| *Cel-pgp-14* | *Caenorhabditis elegans* | NM_077727.6 |
| *Hco-pgp-2* | *Haemonchus contortus* | AF003908 |
| *Hco-pgp-3* | *Haemonchus contortus* | JX430936 |
| *Hco-pgp-9.1* | *Haemonchus contortus* | JX430937 |
| *Hco-pgp-16* | *Haemonchus contortus* | JX430941 |
| *Peq-pgp-16* | *Parascaris equorum* | JX308231.1 |
